# Pathogenesis and Diagnostic Approaches of Avian Infectious Bronchitis

**DOI:** 10.1155/2016/4621659

**Published:** 2016-02-03

**Authors:** Faruku Bande, Siti Suri Arshad, Abdul Rahman Omar, Mohd Hair Bejo, Muhammad Salisu Abubakar, Yusuf Abba

**Affiliations:** ^1^Department of Veterinary Pathology and Microbiology, Faculty of Veterinary Medicine, Universiti Putra Malaysia (UPM), 43400 Serdang, Selangor, Malaysia; ^2^Department of Veterinary Services, Ministry of Animal Health and Fisheries Development, PMB 2109, Usman Faruk Secretariat, Sokoto 840221, Sokoto State, Nigeria; ^3^Laboratory of Vaccine and Immunotherapeutics, Institute of Bioscience, Universiti Putra Malaysia (UPM), 43400 Serdang, Selangor, Malaysia

## Abstract

Infectious bronchitis (IB) is one of the major economically important poultry diseases distributed worldwide. It is caused by infectious bronchitis virus (IBV) and affects both galliform and nongalliform birds. Its economic impact includes decreased egg production and poor egg quality in layers, stunted growth, poor carcass weight, and mortality in broiler chickens. Although primarily affecting the respiratory tract, IBV demonstrates a wide range of tissues tropism, including the renal and reproductive systems. Thus, disease outcome may be influenced by the organ or tissue involved as well as pathotypes or strain of the infecting virus. Knowledge on the epidemiology of the prevalent IBV strains in a particular region is therefore important to guide control and preventions. Meanwhile previous diagnostic methods such as serology and virus isolations are less sensitive and time consuming, respectively; current methods, such as reverse transcription polymerase chain reaction (RT-PCR), Restriction Fragment Length Polymorphism (RFLP), and sequencing, offer highly sensitive, rapid, and accurate diagnostic results, thus enabling the genotyping of new viral strains within the shortest possible time. This review discusses aspects on pathogenesis and diagnostic methods for IBV infection.

## 1. Introduction

Infectious bronchitis (IB) causes significant economic losses to the poultry industry worldwide [1, 2]. The disease was first identified in North Dakota, USA, when Schalk and Hawn reported a new respiratory disease in young chickens [3]. Since then, IBV has been recognized widely, especially in countries with large commercial poultry populations. Apart from respiratory infections, IB affects the kidney and reproductive tract, causing renal dysfunction and decreased egg production, respectively. Although the disease first was believed to occur primarily in young chickens, however, chickens of all age are also susceptible [1].

## 2. Aetiology and Molecular Biology

Infectious bronchitis is caused by infectious bronchitis virus (IBV), a single stranded positive sense, enveloped RNA virus of 27–32 kb length [4]. The virus has been classified under the* Gammacoronavirus* genus in the family Coronaviridae, order Nidovirales. Like other members of coronavirus family, the IBV genome is composed of structural and nonstructural proteins. Structural proteins include the spike [S] glycoprotein, envelope [E], matrix [M], and nucleocapsid [N]. These proteins together play different roles in viral attachment, replication, and inducing clinical disease. Of major structural proteins, the M protein is the most abundant transmembrane protein, which play vital role in coronavirus assembly through interaction with viral ribonucleocapsid and spike glycoprotein [5, 6]. IBV E protein is, however, scant and contains highly hydrophobic transmembrane N-terminal and cytoplasmic C-terminal domains. Studies have shown that the E protein is localized to the Golgi complex in IBV infected cells and is integrally associated with viral envelope formation, assembly, budding, ion channel activity, and apoptosis [7, 8]. Similar to other coronaviruses, the phosphorylated 409 amino acid of IBV-N protein is highly conserved between amino acid residues 238 and 293 [9]. IBV-N protein binds with the genomic RNA to form a helical ribonucleoprotein complex (RNP), thus aiding transcription, replication, translation, and packaging of the viral genome during replication [10]. The S1 portion of the spike glycoprotein plays important role in the attachment and entry of the virus into the cell via sialic acid receptors and has been considered as the determinant for viral diversity and immune protection [11]. This protein has been targeted for genotypic characterization as well as recombinant IBV serotypes vaccines [6, 12, 13].

## 3. Pathogenesis

Infectious bronchitis virus infects primarily the respiratory system. However, some variants and several field isolates affect the reproductive, renal, and digestive systems of chickens. Disease pathogenesis differs according to the system involved, as well as the strain of the virus [1].

### 3.1. Host Susceptibility

Although domestic fowl (*Gallus gallus*) and pheasant (*Phasianus* spp.) are considered to be natural hosts for IBV [14], other IBV-like coronaviruses have been identified in nondomestic avian species including pheasant, peafowl, turkey, teal, geese, pigeon, penguins quail, duck, and Amazon parrot [15–18]. Antigenic similarities between turkey coronavirus (TCoV) and avian infectious bronchitis virus (AIBV) have also been demonstrated [19]. Antibodies to IBV have been demonstrated in humans with close contact to poultry, but the virus has not been reported to cause human clinical disease [20].

### 3.2. Age and Breed Predisposition

Chickens of all ages and breed types are susceptible to IBV infection, but the extent and severity of the disease is pronounced in young chicks, compared to adults. Similarly, resistance to infection was suggested to increase with increasing age [21]. Experimental evidence suggests that line C white leghorn chickens are more resistant to M41 IBV challenge, compared to line 151, although both lines had similar virus shedding rate [22, 23], perhaps influenced by genetic polymorphism in the chicken major histocompatibility complex (MHC), as observed between B^*∗*^15, B^*∗*^13, or B^*∗*^21 chicken haplotypes [24].

### 3.3. Receptor and Entry

The IBV receptor-binding domain (RBD) in the S1-spike plays a major role in attachment of the virus to host cells [25, 26]. Thus, variation in the S1 glycoprotein partly determines tissue tropism and virulence [27, 28]. IBV affects trachea, kidney, and reproductive tract through interaction of S1 glycoproteins RBD (AAs 19–69 in M41) with *α*-2,3-sialic acid receptors on the surface of the cells [29, 30]. In addition to the sialic acid receptor, attenuated Baudette-IBV strain has been shown to interact with a putative heparan sulfate- (HS-) binding site that might contribute to its wide host range [31]. Following viral attachment, conformational changes occurring in the S1 glycoprotein mediate the membrane fusion activity of the S2 carboxylic acid terminal of the spike glycoprotein [1]. Subsequently, IBV enters the cell and releases its nucleocapsid into the cell's cytoplasm, thus triggering replication, virus budding, and release [32].

### 3.4. Infection and Transmission

The virus is transmitted via the respiratory secretions, as well as faecal droplets from infected poultry. Contaminated objects and utensils may aid transmission and spread of the virus from one flock to another. Evidence of virus was shown in trachea, kidney, and Bursa of Fabricius 24 hrs following aerosol transmission [33]. The nature of IBV persistence remains to be elucidated; however, detection of the virus in the caecal tonsils (up to 14 weeks) and from faeces (20 weeks) after infection might suggest a role of faecal shedding in viral transmission and persistence [34].

### 3.5. Incubation Period

Generally the short incubation period for IBV varies with infective dose and route of infection. For example, while infection via the tracheal route may take a course as short as 18 hours, ocular inoculation leads to an incubation period of 36 hours [33].

### 3.6. Clinical Course and Manifestations

**Figure 1:**
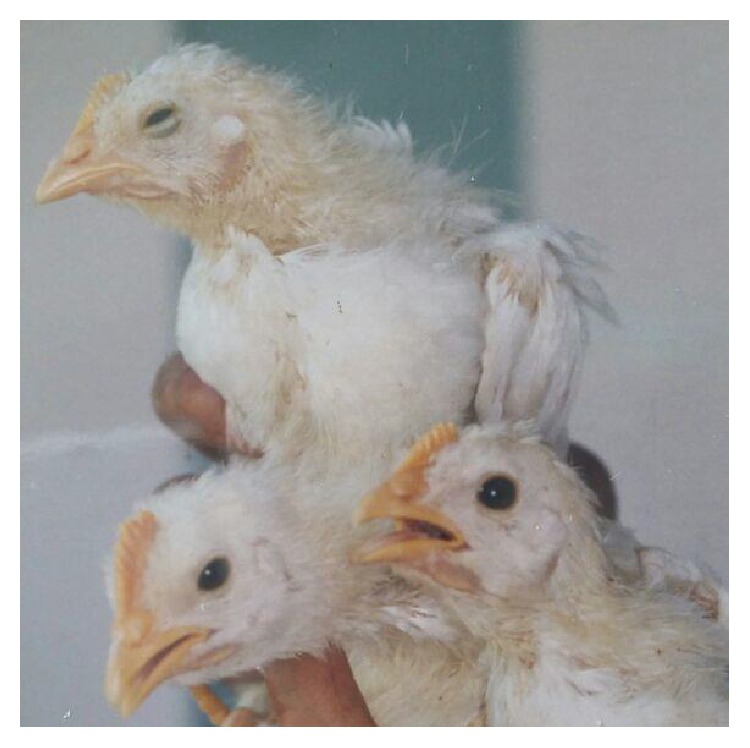
Dullness exhibited in chickens infected following experimental infection with IBV (courtesy: Siti Suri Arshad).

In the host, initial infection occurs at epithelia of Harderian gland, trachea, lungs, and air sacs. The virus then moves to the kidney and urogenital tract, to establish systemic infection [33, 35]. In this regard, the severity and clinical features of IB depend on the organ or system involved. Infection of the respiratory system may result in clinical signs such as gasping, sneezing, tracheal rales, listlessness, and nasal discharges. Affected birds appeared listless and dull with ruffled feathers (Figure 1). Other signs may include weight loss and huddling of birds together under a common heat source [33].

**Figure 2:**
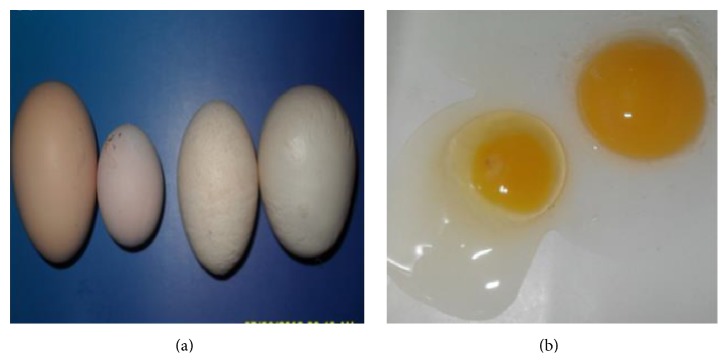
Irregularity in the shape and sizes of eggs from natural IBV infected breeder chickens (a). Watery albumen from IBV infected chicken ((b) left) compared to normal egg ((b) right).

Other clinical outcomes associated with IB infection include frothy conjunctivitis, profuse lacrimation, oedema, and cellulitis of periorbital tissues. Infected birds may also appear lethargic, with evidence of dyspnoea and reluctance to move [36]. Nephropathogenic IBV strains are most described in broiler-type chickens. Clinical signs include depression, wet droppings, and excessive water intake. Infection of reproductive tract is associated with lesions of the oviduct, leading to decreased egg production and quality. Eggs may appear misshapen, rough-shelled, or soft with watery egg yolk (Figure 2). Unless effective measures are instituted, decline in egg production does not return to normal laying, thus contributing to high economic loss [1, 37].

### 3.7. Gross and Histopathology

**Figure 3:**
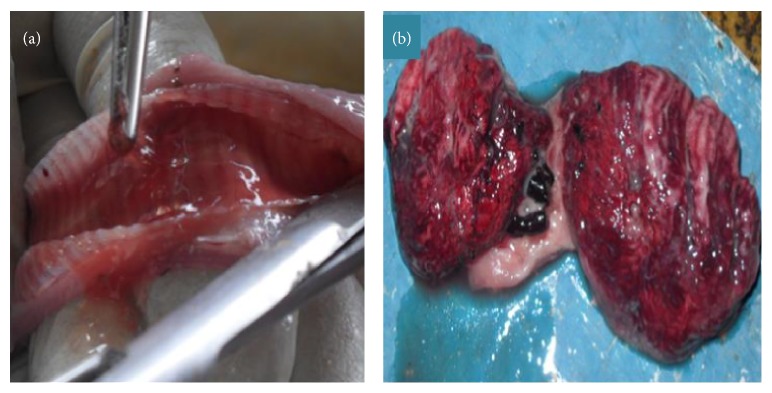
Gross lesions observed on respiratory organs of chicken naturally infected with IBV. Presence of mucoid secretion, congestion, and hyperaemia in the trachea (a); mild focal areas of lung consolidation (b).

Pathological changes observed grossly at necropsy include congestion and oedema of tracheal mucosa and extrapulmonary bronchi (Figure 3) [38, 39].

**Figure 4:**
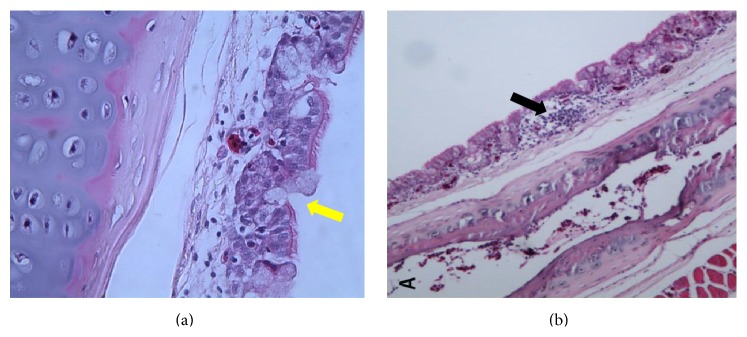
Histopathological changes in the trachea of naturally IBV infected chicken. Note: the marked infiltration of lymphocytes within the epithelia (black arrow (b)) and evidence of mucosal secretions of goblet cells (yellow arrow (a)).

Histopathological changes include loss of cilia, oedema, rounding and sloughing of epithelial cells, and infiltration by lymphocytes (Figure 4). Presence of Russell bodies in Harderian cells has been observed following infection with H120 IBV serotype [40].

**Figure 5:**
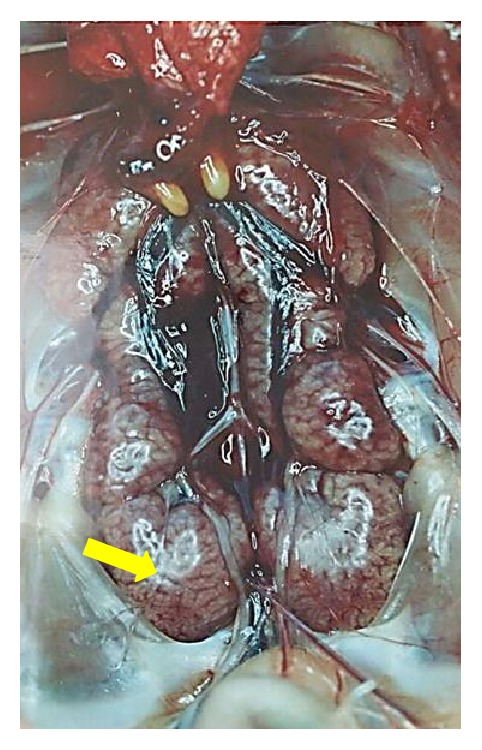
Gross lesions in kidney of chicken following experimental infection with a nephropathogenic infectious bronchitis virus. Note: swelling and congestion of the kidney (arrow) (courtesy: Siti Suri Arshad).

Nephropathogenic IBV strains cause nephritis characterized by swelling and congestion of the kidney (Figure 5), sometimes with pallor of ureters that contain urate deposits. Coinfection with bacterial pathogens such as* E. coli* may lead to a more complex outcome, usually associated with high morbidity and mortality. Similarly, infection with nephropathogenic IBV strains may result in pale, swollen, and mottled kidneys [39, 41]. Histological findings include interstitial nephritis, tubular degeneration, and infiltration by heterophils. In some cases, necrotic and dilated tubules are filled with urates and casts [33]. Experimental studies have shown that IBV-T-strain causes necrosis of the proximal convoluted tubule and distension of distal convoluted tubule. In addition, necrotic foci, heterophils, and lymphocytes are observed in the interstitial spaces. Oedema of Bowman's capsule and granulocytic infiltration has been reported in the collecting ducts and spheroids [42, 43].

**Figure 6:**
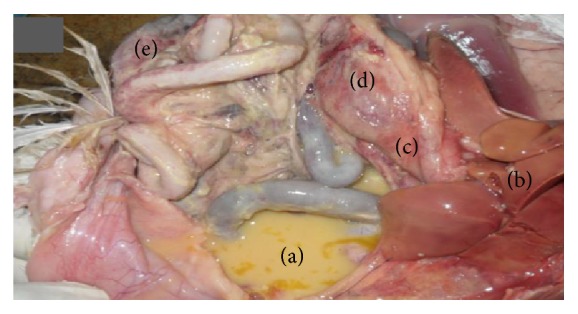
Chicken showing natural IBV infection. Accumulation of egg yolk in abdominal cavity (a); slightly enlarged, pale, friable liver (b) and multiple petechial haemorrhages on the serosal surfaces of proventriculus (c), gizzard (d), and small intestine (e).

**Figure 7:**
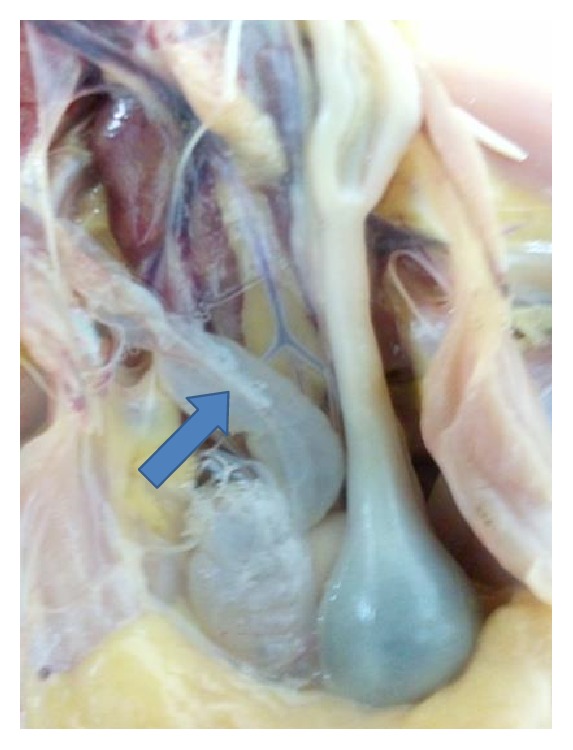
Cystic oviduct in 11-week-old chicken experimentally infected with a CR88 infectious bronchitis virus strain. Note the distention of the entire oviduct and fluid accumulation (arrow).

When the reproductive system is affected, there may be nonpatent and hypoglandular oviduct, especially in severely affected chickens [43, 44]. Large accumulation of yolk fluid may be seen in the abdominal cavity (Figure 6), often associated with bacterial infection in laying hens [45, 46]. Cystic oviduct has also been observed in young layers following infection with certain IBV strains (Figure 7).

### 3.8. Morbidity and Mortality

Morbidity due to IBV infection can reach up to 100%. Mortality rate may range from 25 to 30% in young chicks but may increase to 80% as a result of factors that are host-associated (age, immune status), virus-associated (strain, pathogenicity, virulence, and tissue tropism), or environmental (cold and heat stresses, dust, and presence of ammonia). Secondary bacterial infections (*E. coli*) or coinfection with immunosuppressive viruses such as Marek's disease virus, infectious bursal disease virus (IBDV) [33, 47, 48], may worsen the outcomes of IBV infection. Generally, nephropathogenic IBV strain causes high mortality, compared with strains infecting only the respiratory or reproductive systems [49].

## 4. Diagnosis

Conventional and more advanced methods have been used for the diagnosis of IBV infection. The choice of one test over another is guided by type of sample, availability of test materials and facilities, test reporting time, purpose of the test, and whether the test is carried out in the field or at the laboratory. Selected testing procedures are discussed below.

### 4.1. Serology

In the past, serological assays such as virus neutralization (VN) and haemagglutination inhibition (HI) were used widely for detecting and serotyping IBV strains. These tests also have been used to measure flock protection following vaccination [50, 51]. Serotype-specific antibodies usually are detected using HI, even though the HI test is less reliable [51]. On the other hand, ELISA assays are more sensitive and easily applied for field use and in monitoring antibody response following vaccination or exposure. However, emergence of different IBV serotypes that do not cross-react with commonly available antisera generally made serological tests less applicable and nonconclusive in classifying new or emerging IBV isolates [52, 53].

### 4.2. Virus Isolation and Identification

Virus isolation has been the gold standard for the diagnosis of IBV [54, 55]. Taking samples during early onset of the disease and ensuring the right sampling techniques are important keys for successful isolation of IBV. To support successful virus isolation from swabs, recommended to place swab sample in buffered solutions of media or PBS before transporting them to the laboratory. If tissue samples are to be collected, recommended tissues are trachea, kidney, proventriculus, tonsil, and oviduct. Tissue samples must be collected aseptically from scarified chickens or immediately upon death, placed in sterile, tightly sealed plastic specimen bags, and transported to the laboratory on ice for further processing [56]. The stringent technique requirements and factors, such as the time required for several passages of virus in egg or cell culture, limit the use of virus isolation as a diagnostic method of choice for IBV infection. Notwithstanding, different laboratories use various isolation methods, as described below.

#### 4.2.1. Embryonated Chicken Egg

**Figure 8:**
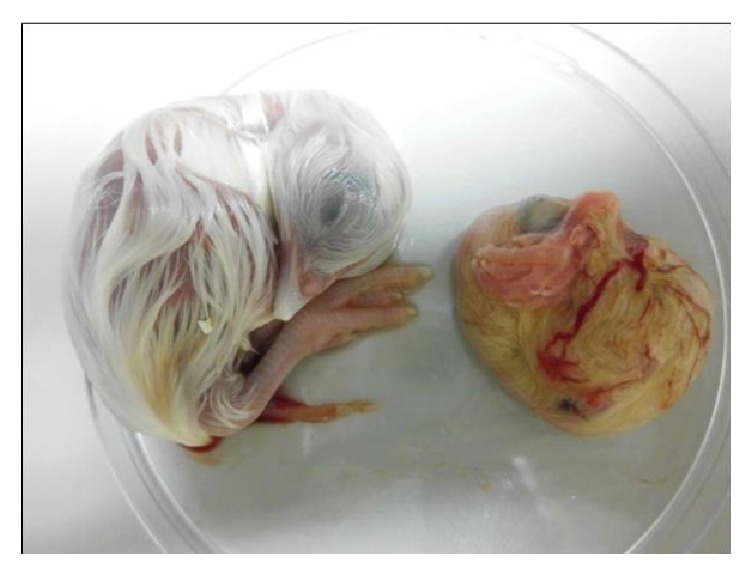
Embryo development at 17 days old following inoculations with IBV-CR88 strain. Note evidence of dwarfism and curling of the toes in IBV infected embryo (right) compared to a noninfected control embryo (left).

Most IBV strains grow well when inoculated into the allantoic cavity of a 9–11-day-old chicken embryo. Clinical samples from tracheal swab, broth, or tissue homogenate (10% w/v) are inoculated into the allantoic cavity of specific pathogen-free eggs and incubated at 34–37°C, after inoculation. Eggs are candled daily to monitor embryo viability; death within 24 hrs is considered nonspecific. After 48–72 hrs, allantoic fluid (AF) is harvested from representative eggs that were chilled overnight and tested for the presence of IBV using serological tests or RT-PCR assay. Sometimes the allantoic fluid needs to be subjected to several passages to allow the virus to adapt and replicate to high titre, thus increasing the period that is needed to obtain results. The latter may vary among viral strains [54]. After 5–7 days, inoculated eggs are opened and observed for characteristic IB lesions such as curling and dwarfism of the infected embryo (Figure 8). It is important to note that such findings are suggestive, but not pathognomonic [57].

#### 4.2.2. Cell Cultures

Isolation of IBV has been attempted in various primary and secondary cells, such as chicken embryo kidney fibroblast and Vero cells, respectively [58, 59]. Infected cultures are characterized by rounding, development of syncytia, and subsequent detachment from the surface of the plate [59]. A major limitation of cell culture methods for IBV isolation is that not all strains of IBV are easily adapted in cell culture. Even for some cell culture adaptable IBV (M41, Iowa 97, and NZ) strains, growth of the virus often requires primary isolation in embryonated eggs and several passages, prior to adaptation. In some cases, attempts to grow IBV in various cell lines either failed or resulted in very low viral titre [58].

#### 4.2.3. Organ Cultures

Tracheal organ culture (TOC) can be used to propagate both embryo-adapted and non-embryo-adapted IBV strains. TOC is prepared from tracheal rings of 20-day-old chicken embryo. The tracheal rings are maintained in a roller bottle and infected with IBV-suspected samples. The culture is observed microscopically for evidence of ciliostasis under light microscope. Complete impairment of ciliary activity usually is considered as a positive culture [60]. Successful growth of IBV has been demonstrated in organ cultures derived from kidney, intestine, proventriculus, and oviduct. However, susceptibility of these organs to IBV can be influenced by the strain of the virus and the amount of virus presence in the sample (infective dose). While a study suggested the universality of using kidney, bursa, and proventriculus in growing IBV, a poor result was obtained when IBV was propagated in cultures derived from different intestinal segments [61]. An advantage of this method includes easy titration and serotyping of IBV, since no virus adaptation is required [62]. Possible constraints include lack of affinity of some IBV strain for some organ cells and difficulty in differentiating ciliostasis arising from other viruses, such as Newcastle disease virus and avian adenovirus [33].

### 4.3. Electron Microscopy

**Figure 9:**
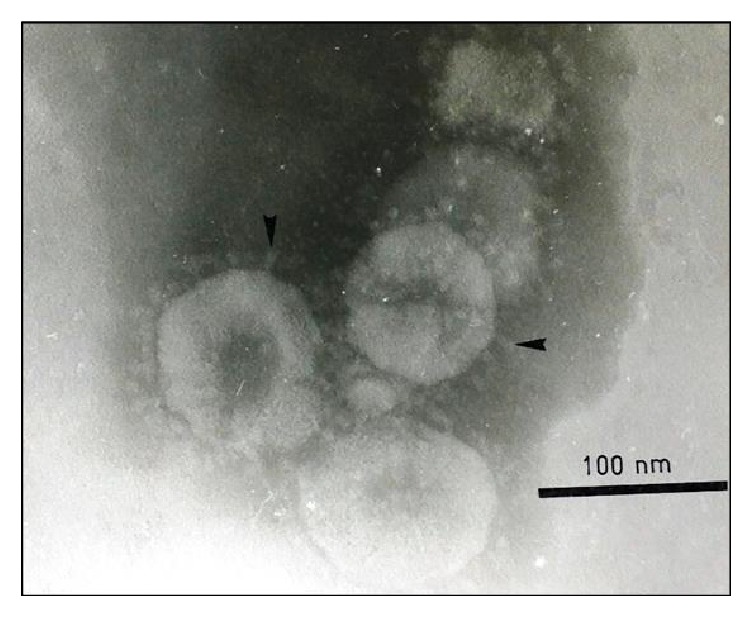
Negative staining electron microscope showing spherical shape of virus with typical spike projections (arrow) surrounding the virion of avian infectious bronchitis virus (courtesy: Siti Suri Arshad).

Electron microscopy provides a direct means of detecting and identifying IBV in biological samples based on morphological characteristics of coronavirus. Positive cultures are confirmed based on the presence of coronavirus-like pleomorphic structures with spike projections, following negative staining with phosphotungstic acid (Figure 9). Importantly, the shape and diameter (120 nm) of the virus are taken into consideration when making diagnostic judgements. Apart from the negative staining method, transmission electron microscopy (TEM) is also a useful tool which enables the visualization of virus-like particles in infected cells [59, 63]. However, this method is often applied to understand viral attachment and entry into the cell but is not a specific diagnostic test [35].

### 4.4. Immunohistochemistry

Immunoperoxidase and immunofluorescence are two important histochemistry methods for detection and confirmation of IBV antigen from infected tissue and/or cells. These methods work based on antigen-antibody reactions [64, 65]. Immunoperoxidase methods such as the avidin-biotin complex (ABC) have been used successfully to localize IBV antigen in tissue samples [66]. Likewise, indirect immunofluorescent assay is the most frequently used fluorescent technique [66, 67].

### 4.5. Molecular Diagnostic Assays

In view of their increased sensitivity and reduced reporting time, molecular methods, such as Reverse Transcriptase Polymerase Chain Reaction (RT-PCR), real-time PCR, Restriction Fragment Length Polymorphism (RFLP), and genome sequencing, have nearly replaced conventional serology and virus cultivation methods of IBV diagnosis [68, 69].

#### 4.5.1. RT-PCR Methods

**Figure 10:**
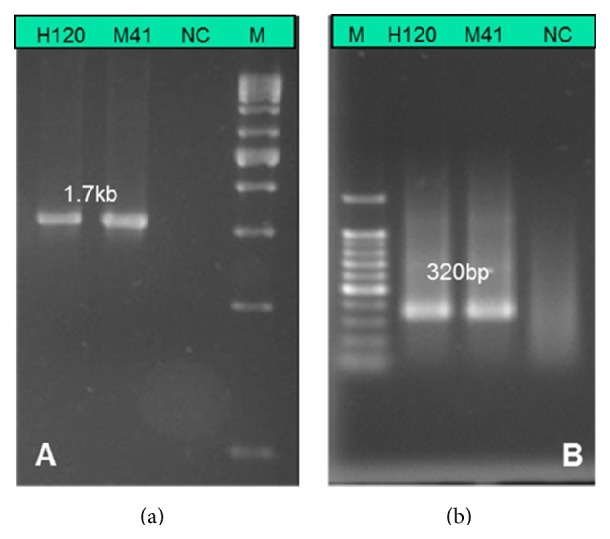
Electropherogram showing 1.7 kb RT-PCR amplified S1 genes from vaccine (H120) and virulent (M41) IBV strains (a) compared to a 320 bp RT-PCR amplified N-gene (b) of H120 and M41 IBV serotypes. Lane M = 1 kb molecular ladder (a) and 100 bp ladder (b); lane NC: negative control (no template control).

This approach uses viral RNA, amplified either directly (one-step RT-PCR) or following cDNA synthesis (two-step RT-PCR). An RT-PCR assay was designed and introduced in 1991 for detecting the IBV-S2 gene [70]. Subsequently, general and serotype-specific RT-PCR assays were designed to target different regions and/or fragments (Figure 10) in the IBV viral genome [71–73]. The UTR and N-gene-based RT-PCR are used for universal detection, because of the conserved nature of the target region in many IBV serotypes [68, 71]. A pan-coronavirus primer, targeting a conserved region of different coronavirus isolates, could also be used in one-step RT-PCR amplification of IBV strains [55]. However, amplification and sequencing of the S1 gene provide a reliable means for genotypic classification of new IBV strains [74]. A serotype-specific PCR assay has been designed to enable differentiation of Massachusetts, Connecticut, Arkansas, and Delaware field isolates [73].

#### 4.5.2. Restriction Fragment Length Polymorphism (RFLP)

This is an IBV genotyping method carried out to differentiate different known strains of IBV and to identify new variants following RT-PCR amplification [75]. Full-length sequence of IBV S1 glycoprotein could be targeted for amplification and enzymes analysis [72, 76]. RFLP allows differentiation of various known IBV strains, based on their unique electrophoresis banding patterns defined by restriction enzyme digestion [72, 77]. The assay was found to be comparable with traditional virus neutralization assay, although some strains such as the Gray and JMK strains were reportedly difficult to differentiate using arrays of restriction enzymes, thus limiting the universal application of this method [72].

#### 4.5.3. Real-Time RT-PCR and Other Forms of PCR Assays

For increased test sensitivity and specificity, real-time RT-PCR assays [78, 79] have been introduced for detecting IBV. Apart from detection, it is possible to quantify IBV viral load from tissue and/or clinical samples by real-time RT-PCR assays based on viral copy number or fold changes [80, 81]. Likewise, differentiation of Massachusetts from non-Massachusetts is possible by real-time RT-PCR assay targeting S1 glycoprotein gene [79, 82]. Recently, a high resolution melt curve analysis (HRM) was also developed to allow differentiation of field from vaccine IBV strains as well as for rapid and sensitive detection of recombinant variants [83, 84]. Meir et al. [85] reported that real-time RT-PCR was comparable to virus isolation and one or two times more sensitive in detecting M41 IBV than ordinary N-gene and S1 gene specific RT-PCR assays. On the other hand, real-time RT-PCR was tenfold more sensitive compared to virus isolation and 30- or 40-fold compared to N-gene or S1 gene-based RT-PCR, respectively. The authors, however, reported variations in sensitivity when either N-gene or S1 genes were targeted as well as when different samples are used for viral amplification. Other forms of PCR methods used in detecting IBV include nested PCR [68]; multiplex PCR [86]; and reverse transcription loop-mediated isothermal amplification (RT-LAMP) [87]. While these methods are more sensitive than standard RT-PCR, they are more expensive as well and might be beyond the financial capacity of many producers.

#### 4.5.4. Sequence and Phylogenetic Analyses

**Figure 11:**
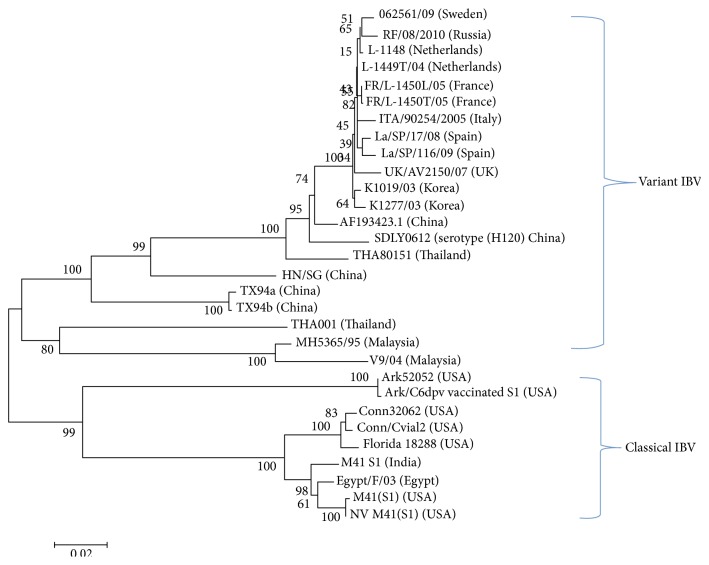
Neighbour joining phylogenetic analysis based on nucleotide acid sequence of S1-spike gene of classical and variant IBV strains identified in different countries. The tree was drawn with MEGA5 software using 1000 bootstrap replicates.

For genotyping, S1 gene usually is amplified using RT-PCR, sequenced, and subjected to bioinformatics analyses [88, 89]. Following S1 gene sequencing, isolates are characterized through bioinformatics analyses based on their phylogenetic relatedness with reference sequences available in sequence databases such as the NCBI, EMBL, and DDBJ (Figure 11). Lack of method standardization among laboratories, particularly with respect to the S1 gene segment length that is used in phylogenetic analysis, limits genotyping to some extent. Currently, molecular methods such as next generation sequencing (NGS) have been introduced to sequence whole genomes within limited periods of time, though this approach has been used only in the laboratory.

## 5. Differential Diagnosis

Several respiratory diseases, such as Newcastle disease (ND), infectious laryngotracheitis, infectious coryza, avian metapneumovirus (aMPV), and avian influenza (AI), may produce clinical signs similar to avian infectious bronchitis. However, certain clinical features, including neurological signs and diarrhoea in ND, high mortality in AI, and pronounced head swelling in coryza, are uncommon in IBV infection and thus may be used in ruling out or arriving at narrowed tentative differential list [33, 90].

## 6. Conclusion

Ever since the first identification of IBV in 1930s, the poultry industry has suffered a growing number of emerging IBV serotypes. Importantly, the newly evolved strains have been favoured by selection pressure, mutation, and/or recombinations, thus allowing them to avoid detection, evade host immune response, and cause diverse pathological outcomes. Lack of effective diagnostic methods and vaccines that could easily tackle the menace caused by multiple IBV serotypes is partly blamed for the serious economic losses as results of infectious bronchitis disease. Conventional detection assays such as virus neutralization and virus isolation have been used extensively, but, due to lack of sensitivity and specificity of serological assays and laborious nature of virus isolation methods, these assays have gradually been replaced by the new sensitive and specific assays such as RT-PCR, RFLP, and qRT-PCR that enable rapid genotyping and identification of new IBV strains. However, there is a need for standardization across laboratories with respect to the type and length of target gene to be considered for genotyping so as to ensure common understanding of genotype distributions in order to guide vaccine selection for prevention and control.
